# The Role of Posttraumatic Hypothermia in Preventing Dendrite Degeneration and Spine Loss after Severe Traumatic Brain Injury

**DOI:** 10.1038/srep37063

**Published:** 2016-11-11

**Authors:** Chuan-fang Wang, Cheng-cheng Zhao, Gan Jiang, Xiao Gu, Jun-feng Feng, Ji-yao Jiang

**Affiliations:** 1 Department of Neurosurgery, Ren Ji Hospital, School of Medicine, Shanghai Jiao Tong University, Shanghai 200127, People’s Republic of China; 2 Shanghai Institute of Head Trauma, Shanghai 200127, People’s Republic of China; 3 Department of Pharmacology, Institute of Medical Science, Shanghai Jiaotong University School of Medicine, Shanghai 200127, People’s Republic of China

## Abstract

Posttraumatic hypothermia prevents cell death and promotes functional outcomes after traumatic brain injury (TBI). However, little is known regarding the effect of hypothermia on dendrite degeneration and spine loss after severe TBI. In the present study, we used thy1-GFP transgenic mice to investigate the effect of hypothermia on the dendrites and spines in layer V/VI of the ipsilateral cortex after severe TBI. We found that hypothermia (33 °C) dramatically prevented dendrite degeneration and spine loss 1 and 7 days after CCI. The Morris water maze test revealed that hypothermia preserved the learning and memory functions of mice after CCI. Hypothermia significantly increased the expression of the synaptic proteins GluR1 and PSD-95 at 1 and 7 days after CCI in the ipsilateral cortex and hippocampus compared with that of the normothermia TBI group. Hypothermia also increased cortical and hippocampal BDNF levels. These results suggest that posttraumatic hypothermia is an effective method to prevent dendrite degeneration and spine loss and preserve learning and memory function after severe TBI. Increasing cortical and hippocampal BDNF levels might be the mechanism through which hypothermia prevents dendrite degeneration and spine loss and preserves learning and memory function.

Traumatic brain injury (TBI) is a leading cause of mortality and disability. According to public epidemiological statistics, nearly 250 per 100,000 persons experience TBI each year; thus, this injury represents a serious public health problem worldwide^1^,^2^. TBI not only induces a primary insult but also initiates secondary damage that causes extensive cell death and degeneration in the whole brain^3^,^4^. TBI patients may suffer from enduring neurological deficit such as paralysis, dementia, memory loss and long-term coma.

In addition to cell death, extensive dendrite and spine degeneration as well as a significant reduction in the number of synapses occur after TBI^5^. TBI has been shown to cause structural and functional deficits of synapses, thereby contributing to motor disorder and memory impairment^6^,^7^. Effective dendrite protection and spine preservation strategies, such as the intraperitoneal injection of the brain-derived neurotrophic factor (BDNF) analogue 7,8-dihydroxyflavone, can improve behavioural outcomes after TBI in mice^8^.

Posttraumatic mild hypothermia is an effective technique for reducing cerebral metabolism and oxygen consumption, diminishing cytotoxic oedema, stabilizing the blood-brain barrier (BBB), and promoting the survival rate after TBI; furthermore, it is widely applied in clinical practice^9^. Several animal studies have also revealed that posttraumatic hypothermia can prevent cell death and axonal degeneration after TBI^10^,^11^. In previous study, we found that mild hypothermia significantly improved the outcomes of severe TBI compared with that of a normothermia group after a one-year follow-up assessment^12^. However, the effects of posttraumatic hypothermia on TBI-induced dendrite degeneration and spine loss during the early phase of this condition remain unknown.

In the present study, we used thy1-GFP transgenic mice to establish a controlled cortical impact (CCI) injury model. We than applied therapeutic mild hypothermia treatment (33 °C) to investigate whether posttraumatic hypothermia affects dendrite protection and spine preservation at 1 and 7 days after TBI and determine the possible underlying mechanisms of this effect.

## Methods

### Animals

Professor Nan-jie Xu (Department of Anatomy, Histology and Embryology, Neuroscience Division, Shanghai Jiao Tong University School of Medicine, Shanghai, China) provided 150 adult (8- to 10-week-old) male thy1-GFP transgenic mice for use in this experiment. All the animals were allowed free access to water. Food was withheld overnight before surgery. The animals were housed under a 12-h/12-h light/dark cycle at a controlled temperature and humidity and were randomly divided into three groups: the TBI with normothermia group (TNG; n = 50, 37 °C), the TBI with hypothermia group (THG; n = 50, 33 °C) and the sham group (n = 50). The Animal Care and Experiment Committee of the School of Medicine, Shanghai Jiao Tong University approved all the animal procedures, and all experiments were performed in accordance with the guidelines of the National Institutes of Health regarding animal care.

### Controlled cortical impact traumatic brain injury

The animal model of controlled cortical impact (CCI) traumatic brain injury (TBI) was prepared as previously described with some modifications^8^,^13^. In brief, the mice were anaesthetized with 2% isoflurane and endotracheally intubated for mechanical ventilation. A thermal heating blanket was used to maintain the body temperature of the mice at 37 °C. Each mouse was placed in a stereotaxic frame (Stoelting, Varese, Italy) prior to TBI. Following a midline incision, a circular craniotomy (4-mm diameter) was performed on the left side at a central location midway between the central incision and the temporalis muscle and midway between the bregma and lambda. The skullcap was carefully removed without disrupting the underlying dura. Prior to the injury, the impacting piston was controlled at a 15°- to 20° angle to the vertical plane, such that the impacting tip (3-mm diameter) was perpendicular to the exposed cortical surface. The mice (n = 100) were subjected to a severe cortical contusion using an electromagnetically controlled impacting device (PinPoint™ PCI3000 Precision Cortical Impactor™, Hatteras Instruments, Cary, USA); the amount of deformation was set at 1.5 mm, and the piston velocity was set at 3 m/s. In the sham group, the mice received craniotomies without CCI injury.

### Temperature manipulation

The temperature manipulation was initiated immediately after scalp suturing. Temporalis muscle and rectal temperature probes were used to measure brain and body temperatures, respectively. The brain temperatures of the mice in the THG were maintained at 33 °C, and those of the TNG and sham group were maintained at 37 °C. Hypothermia was achieved by immersing the packed mice into ice-cold water and the mice were protected from direct contact with the water. The target temperature was achieved within 30 minutes after CCI injury and maintained for 4 h; the mice were then slowly rewarmed to normothermia over 90 minutes. Twenty mice from each group were sacrificed for western blot analysis (n = 10) and morphological observations (n = 10) at 1 and 7 days after CCI injury. Sixty mice were sacrificed at each time point for a total of 120 mice. The remaining mice (n = 30, ten mice from each group) were subjected to the Morris water maze (MWM) test two weeks after TBI.

### Slice preparation

The mice were deeply anesthetized with 2% isoflurane and then perfused transcardially with 0.9% saline (4 °C), followed by PBS containing 4% paraformaldehyde (4 °C). Brain samples were collected and post-fixed in PBS containing 4% paraformaldehyde overnight at 4 °C. The brains were then dehydrated in 15%, 20% and 30% gradient sucrose solutions for 24 h at 4 °C in the dark. The brain tissues were then cut into 50-μm coronal sections and stored at −20 °C.

### Dendrite morphology analysis and spine quantification

Pyramidal neurons 2 mm adjacent to the edge of the cortical cavity were collected for analysis. For each selected neuron, dendrites were taken and reconstructed (reconstruction thickness, 0.5 μm) at 200× magnification using a Zeiss Axio Observer Z1 instrument and ZEN (blue edition) software (Zeiss, Oberkochen, Germany). The final reconstructed dendrites were obtained using a maximum-intensity projection strategy. The following analysis of the reconstructed dendrites was then performed using NeuronJ^14^ and NeuronStudio^15^,^16^. Ten neurons were analysed for each mouse in the three experimental groups. The dendrite morphology analysis included the number of dendritic branches and their total and average lengths as well as a Sholl analysis. The Sholl analysis was performed at radial intervals of 30 μm.

For spine quantification, the spines located at the main apical dendrite were analysed. Images were captured and reconstructed (reconstruction thickness, 0.05 μm) at 630× magnification (oil immersion objective). The final reconstructed spines were obtained using a maximum-intensity projection strategy. The spine quantification was assessed at 100-μm intervals using NeuronStudio. The spines from ten apical dendrites were studied for each mouse from the three experimental groups.

### Morris water maze

Two weeks after CCI injury, learning and memory functions were evaluated using the Morris water maze (MWM) test. The maze was composed of a round black pool 120 cm in diameter and 50 cm in depth. A black platform 6 cm in diameter and 30 cm in height was placed in the southwest quadrant of the pool. The pool was filled with water at 22 ± 1 °C, and the platform was hidden 1 cm under the water surface. In each trial, mice were released from one of four directions (east, south, west and north) and allowed to swim. Each mouse was allowed 60 s to discover the underwater platform. When the mouse arrived at the platform, it was allowed to rest on the platform for 20 s. If the mouse did not find the platform within 60 s, it was guided to the platform and allowed to remain for 20 s. After each trial, the mice were placed in a dry cage. Each mouse was tested across four trials starting from four different start positions per day for five consecutive days. On the sixth day, each mouse was tested in one trial to assess memory function. Mouse movement was recorded using a video tracking system (DigBehv, Jiliang Software Technology Company, Shanghai, China), and the results (including latency and swimming distance) were collected and calculated for statistical analysis.

### Western blot

Mice were deeply anaesthetized with 2% isoflurane and perfused transcardially with 0.9% saline (4 °C). Brain samples were dissected and lysed using the RIPA lysis buffer system (Santa Cruz, California, USA). Sample lysates were centrifuged at 4 °C, and the protein supernatants were collected and quantified using the BCA protein assay kit (Beyotime, Jiangsu, China). The supernatants were then diluted in 5 × SDS loading buffer and denatured at 100 °C for 5 minutes. Protein samples were electrophoretically separated in a 10% SDS-PAGE gel or 15% SDS-PAGE gel and transferred onto polyvinylidene difluoride membranes (Millipore, Merck KGaA, Darmstadt, Germany). The membranes were then blocked in 5% skim milk at room temperature for 1 h and incubated overnight at 4 °C with primary antibodies. After washing 3 times for 10 minutes, the membranes were incubated with secondary antibodies. Signals were detected using the Immobilon Western Chemiluminescent HRP Substrate (Millipore, Merck KGaA, Darmstadt, Germany). The following primary antibodies were obtained: monoclonal rabbit anti-synaptophysin (1:80,000, Abcam, Cambridge, UK), monoclonal rabbit anti-glutamate receptor 1 (GluR1; 1:5,000, Abcam, Cambridge, UK), monoclonal rabbit anti-postsynaptic density-95 (PSD-95; 1:20,000, Abcam, Cambridge, UK), monoclonal rabbit anti-BDNF (1:1,000, Abcam, Cambridge, UK), polyclonal rabbit anti-TrkB (1:1,000, Abcam, Cambridge, UK) and monoclonal mouse anti-β-tubulin (1:1,000, Cell Signaling Technology, Beverly, MA, USA). The following secondary antibodies were used: goat anti-mouse IgG-HRP (1:5,000, Bioworld Technology, Minnesota, USA) and goat anti-rabbit IgG-HRP (1:5,000, Bioworld Technology, Minnesota, USA).

### Statistical analysis

All the data are presented as the means ± standard errors of the mean (SEMs). Statistical analyses were performed using SPSS 16.0. Statistical significance was determined using a one-way analysis of variance (ANOVA) followed by Tukey’s post hoc test. Differences in which *p* < 0.05 were considered as significant.

## Results

### Posttraumatic hypothermia prevented dendrite degeneration after severe TBI

**Figure 1:**
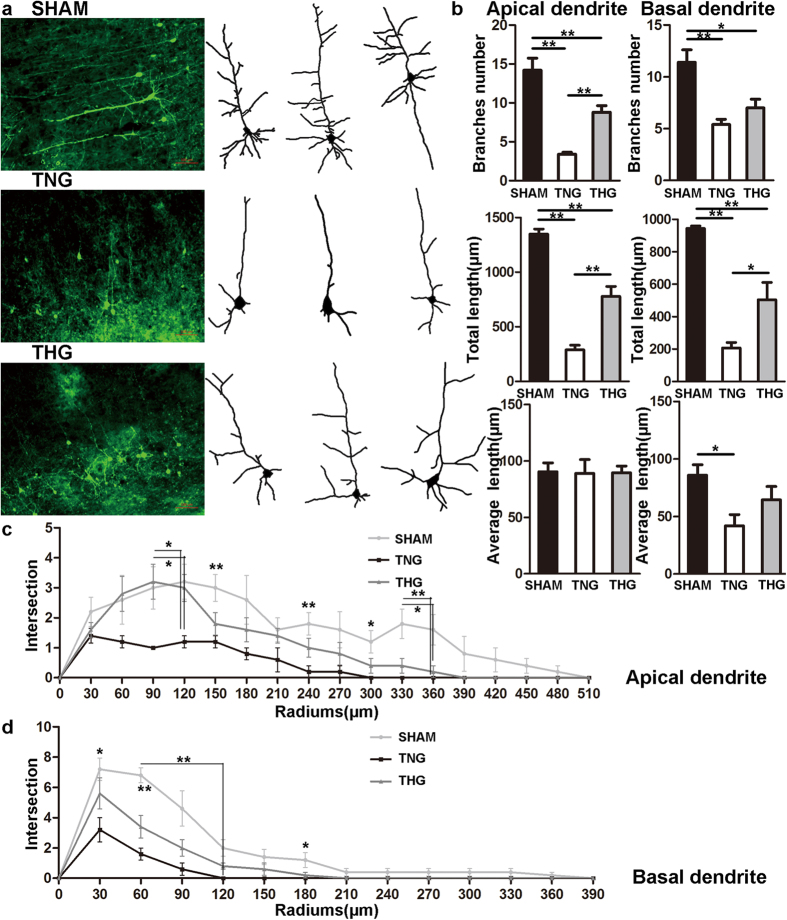
Posttraumatic hypothermia prevented dendrite degeneration 1 day after severe TBI. (**a**) Image data were collected using fluorescence microscope. *Left panel:* Cortical pyramidal neurons within 2 mm of the lesion cavities of the three groups. *Right panel:* Reconstruction of the cortical pyramidal neurons in the three groups. (**b**) Branches number, total length and average length were compared across the three groups. Compared with the TNG, the THG showed increased branch number and total length in apical dendrites as well as increased total length in basal dendrites. (**c,d**) A Sholl analysis was used to assess the observed changes in dendrite complexity. Compared with the TNG, the THG had more intersections 90–120 μm from the neuron soma in the apical dendrites. The data are represented as mean ± SEMs and analysed using one-way analysis of variance (ANOVA) followed by Tukey’s post hoc test, n = 10, *p < 0.05, **p < 0.01, THG or sham versus TNG. TNG, the traumatic brain injury with normothermia group; THG, the traumatic brain injury with hypothermia group.

**Figure 2:**
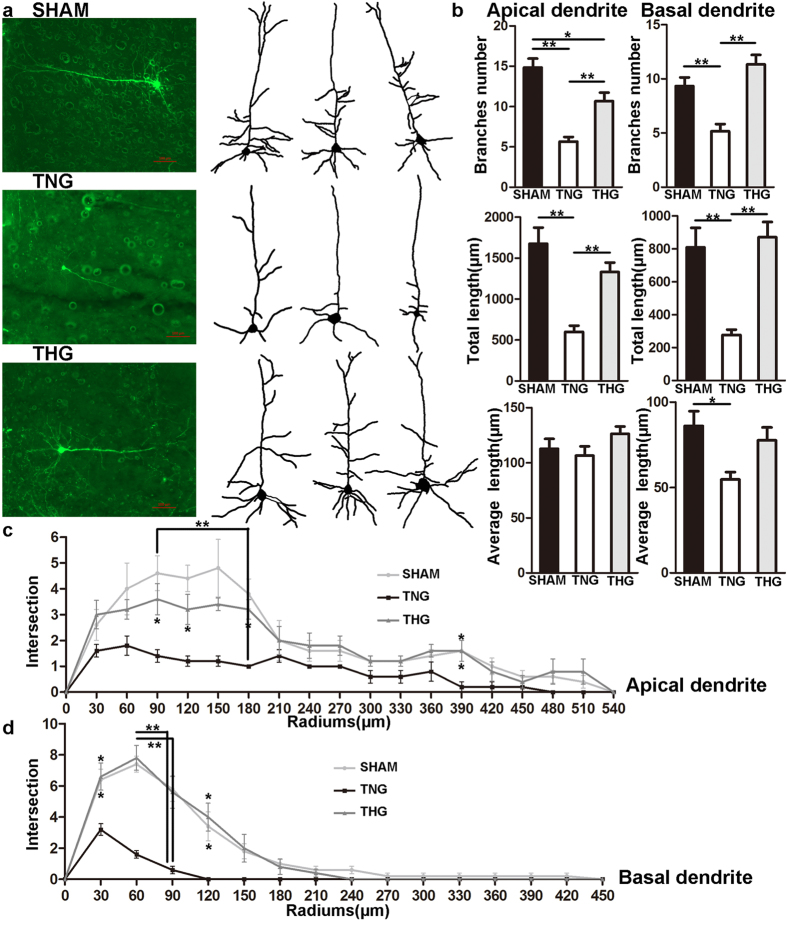
Posttraumatic hypothermia prevented dendrite degeneration 7 days after severe TBI. (**a**) Image data were collected using fluorescence microscopy. *Left panel:* Cortical pyramidal neurons within 2 mm of the lesion cavities of the three groups. *Right panel:* Reconstruction of cortical pyramidal neurons in the three groups. (**b**) Branch number, total length and average length were compared across the three groups. Compared with the TNG, the THG showed increased branch number and total length in apical and basal dendrites. (**c,d**) A Sholl analysis was used to assess the observed changes in dendrite complexity. Compared with the TNG, the THG had more intersections 90–120, 180 and 390 μm from the neuron soma in the apical dendrites and 30–120 μm from the neuron soma in basal dendrites. The data are represented as the means ± SEMs and analysed using a one-way analysis of variance (ANOVA) followed by Tukey’s post hoc test, n = 10, *p < 0.05, **p < 0.01, THG or sham vs. TNG. TNG, the traumatic brain injury with normothermia group; THG, the traumatic brain injury with hypothermia group.

To determine whether posttraumatic hypothermia prevents dendrite branch degeneration in the pericontusion region after severe TBI, we assessed the morphology changes of cortical pyramidal neurons in layer V/VI within 2 mm of the lesion cavity (Figs 1a and 2a).

One day after severe TBI, significant reductions (*p* < 0.01) in the number of dendrite branches and total length were observed in the TNG and THG compared with the sham group (Fig. 1b). Furthermore, the number of apical dendrite branches in the TNG was 3.4 ± 0.2, and the total length of the dendrite branches was 289 ± 42.7 μm; the THG exhibited marked increases in the number of dendrite branches (from 3.4 ± 0.2 to 8.8 ± 1.4, *p* < 0.01) and total length of dendrite branches (from 289 ± 42.7 μm to 777.8 ± 92.3 μm, *p* < 0.01) compared with TNG. However, the average length of apical dendrite branches did not significantly differ among the three groups (Fig. 1b). Posttraumatic hypothermia exerted a significant protective effect on the apical dendrites, although both the number and total length of the dendrite branches did not reach the normal level.

In addition, the basal dendrites of the THG exhibited a marked increase in the total length of dendrite branches (504 ± 106.3 μm) compared with that of the TNG (206.8 ± 33.8 μm, *p* = 0.025), whereas the number of dendrite branches did not significantly differ between these groups (THG vs. TNG, 7 ± 0.8 vs. 5.4 ± 0.5, respectively, *p* = 0.4; Fig. 1b). The TNG (41.7 ± 9.9 μm) exhibited a decrease in the average length of their basal dendrite branches compared with that of the sham group (85.8 ± 9.1 μm, *p* = 0.035; Fig. 1b).

In addition, a Sholl analysis was performed to evaluate the changes in dendrite complexity. As Fig. 1(c,d) shows, the apical and basal dendrites exhibited marked decreases in complexity after severe TBI. The apical dendrites of the THG showed more intersections than those of the TNG within 90–120 μm of the neuron soma (90 μm: *p* = 0.031; 120 μm: *p* = 0.033; Fig. 1c). The basal dendrites of the THG exhibited similar intersections to those observed in the TNG (Fig. 1d).

Seven days after severe TBI, significant reductions (*p* < 0.01) in the number of dendrite branches and their total length were observed in the TNG compared with the sham group, whereas a reduction (*p* = 0.018) was only observed for the number of apical dendrite branches in the THG compared with the sham group (Fig. 2b). The number of basal dendrite branches and their total length did not significantly differ between the THG and sham group. Moreover, the number of apical dendrite branches (from 5.7 ± 0.6 to 10.7 ± 1.1, *p* < 0.01) and the total length of dendrite branches (from 600.5 ± 74.1 μm to 1330 ± 116.1 μm, *p* < 0.01) were significantly increased in the THG compared with the TNG. The average length of the apical dendrite branches did not significantly differ among the three groups (Fig. 2b).

The number of basal dendrite branches and their total length did not significantly differ between the THG and sham group (Fig. 2b). In addition, significant increases in the number of dendrite branches (THG vs. TNG, 11.3 ± 0.9 vs. 5.2 ± 0.7, respectively, *p* < 0.01) and total length (THG vs. TNG, 870.4 ± 93.0 μm vs. 277.3 ± 31.9 μm, respectively, *p* < 0.01) were observed. The TNG (54.6 ± 4.4 μm) exhibited a decreased average basal dendrite branch length compared with the sham group (86.0 ± 8.6 μm, *p* = 0.018; Fig. 2b).

The Sholl analysis showed that the complexity of the apical and basal dendrites did not significantly differ between the THG and sham group (Fig. 2c,d). Moreover, the apical dendrites of the THG showed more intersections than those in the TNG 90–120, 180 and 390 μm from the neuron soma (90 μm, *p* = 0.035; 120 μm, *p* = 0.047; 180 μm, *p* < 0.01; 390 μm, *p* = 0.036; Fig. 2c). The basal dendrites of the THG showed more intersections than those in the TNG 30–120 μm from the neuron soma (30 μm, *p* = 0.010; 60 μm, *p* < 0.01; 90 μm, *p* < 0.01; 120 μm, *p* = 0.037; Fig. 2d).

These results clearly indicate that posttraumatic hypothermia significantly prevented dendrite degeneration at 1 and 7 days after severe TBI. Moreover, the number of basal dendrite branches and the total length of apical and basal dendrite branches reached the normal level 7 days after severe TBI.

### Posttraumatic hypothermia prevented spine loss after severe TBI

**Figure 3:**
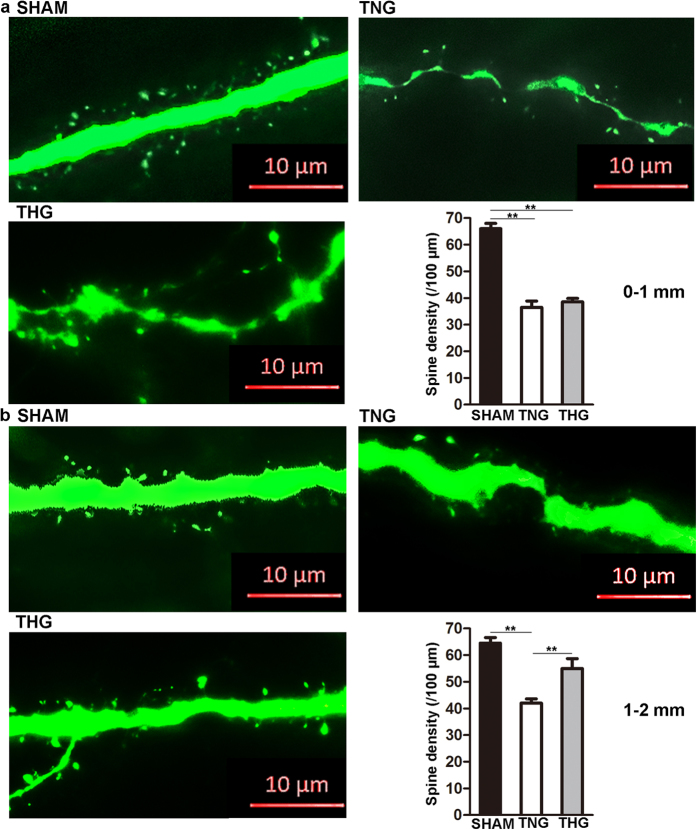
Posttraumatic hypothermia prevented spine loss 1 day after severe TBI. (**a**) Representative images and quantitative analyses of spines (right bottom panel) in the three groups within 0–1 mm from the edge of the lesion cavity. (**b**) Representative images and quantitative analyses of the spines (right bottom panel) of three groups within 1–2 mm from the edge of the lesion cavity. High-resolution images were captured using fluorescence microscopy at a magnification of 630×. The data are represented as the means ± SEMs and analysed using one-way analysis of variance (ANOVA) followed by Tukey’s post hoc test, n = 10, **p < 0.01, THG or sham versus TNG. TNG, the traumatic brain injury with normothermia group; THG, the traumatic brain injury with hypothermia group.

To further investigate whether posttraumatic hypothermia prevents spine loss after severe TBI, we evaluated the apical dendrite spines of the cortical pyramidal neurons in layer V/VI within 2 mm of the lesion cavity. As Fig. 3 shows, the mice underwent a marked spine loss 2 mm from the edge of lesion cavity compared with that of the sham group 1 day after severe TBI (0–1 mm, *p* < 0.01; 1–2 mm, *p* < 0.01). Between 0 and 1 mm, the spine density did not differ between the THG (37.2 ± 1.7/100 μm) and TNG (36.6 ± 1.8/100 μm; *p* = 0.97; Fig. 3a). Between 1 and 2 mm area, the spine density in THG was significantly increased from 37.2 ± 1.7/100 μm to 54.6 ± 2.9/100 μm (*p* < 0.01), whereas that in the TNG remained at a low level, from 36.6 ± 1.8/100 μm to 41 ± 1.6/100 μm (Fig. 3b).

**Figure 4:**
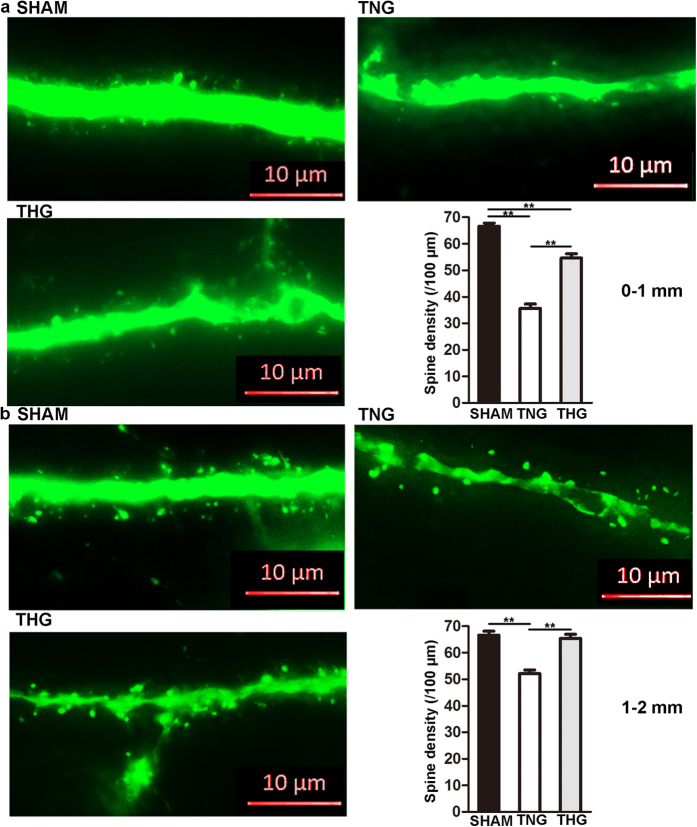
Posttraumatic hypothermia prevented spine loss 7 days after severe TBI. (**a**) Representative images and quantitative analyses of the spines (right bottom panel) of the three groups within 0–1 mm from the edge of the lesion cavity. (**b**) Representative images and quantitative analyses of the spines (right bottom panel) in the three groups within 1–2 mm from the edge of the lesion cavity. High-resolution images were captured using fluorescence microscopy at a magnification of 630×. The data are represented as the means ± SEMs and were analysed using a one-way analysis of variance (ANOVA) followed by Tukey’s post hoc test, n = 10, **p < 0.01, THG or sham vs. TNG. TNG, the traumatic brain injury with normothermia group; THG, the traumatic brain injury with hypothermia group.

Seven days after severe TBI, spine density continued to dramatically decrease in the THG and TNG compared with that of the sham group (*p* < 0.01) in the 0–1 mm area (Fig. 4c). However, the THG (54.6 ± 1.6/100 μm) showed a higher spine density than the TNG (35.6 ± 1.7/100 μm; *p* < 0.01). In the 1–2 mm area, the spine density did not significantly differ between the THG (65.4 ± 1.5/100 μm) and the sham group (66.6 ± 1.5/100 μm; *p* = 0.83), whereas that of the TNG (52.2 ± 1.3/100 μm) was still maintained at a low level (Fig. 4b).

These results show that posttraumatic hypothermia prevented spine loss at both 1 and 7 days after severe TBI.

### Posttraumatic hypothermia improved learning and memory function after severe TBI

**Figure 5:**
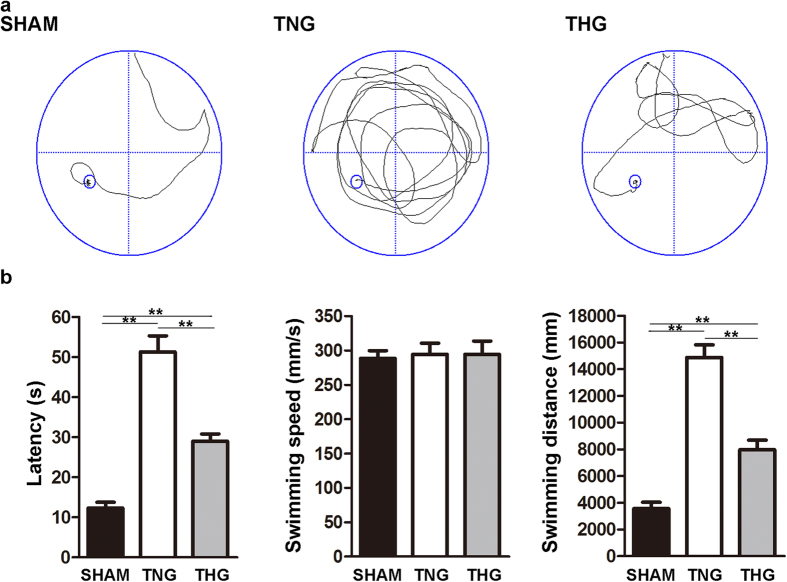
Posttraumatic hypothermia improved learning and memory function recovery after severe TBI. (**a**) Typical swimming patterns of the mice in all three groups on the sixth day of the Morris water maze test. (**b**) The Morris water maze test showed that the latency and the swimming distance of the mice in the THG had significantly decreased compared with those in the TNG 2 weeks after severe TBI. However, swimming speed did not significantly differ among the three groups. The data are represented as the means ± SEM and were analysed using a one-way analysis of variance (ANOVA) followed by Tukey’s post hoc test, n = 10, **p < 0.01, THG or sham vs. TNG. TNG, the traumatic brain injury with normothermia group; THG, the traumatic brain injury with hypothermia group.

The MWM test was performed to assess the effect of posttraumatic hypothermia on learning and memory functions after severe TBI. Latency (the time taken to find the platform), swimming speed and swimming distance were analysed. Figure 5 compares the TNG with the sham group regarding latency (51.28 ± 4 s vs. 12.29 ± 1.47 s, respectively) and swimming distance (14,873 ± 943 mm vs. 3,564 ± 472 mm, respectively), showing that these values were significantly increased on the MWM test six days after severe TBI for the former group (*p* < 0.01). Compared with the corresponding values in the TNG, the latency and the swimming distance of the THG were significantly attenuated (latency, 51.28 ± 4 s vs. 27.36 ± 2.44 s, respectively, *p* < 0.01; swimming distance, 14,873 ± 943 mm vs. 7,967 ± 714 mm, respectively, *p* < 0.01). These findings suggested that hypothermia protected learning and memory function and improved behavioural outcomes after severe TBI. However, the latency and swimming distance in THG remained two-fold greater than the corresponding values in the sham group, indicating that learning and memory functions in the THG did not reach normal levels. In addition, swimming speed did not significantly differ among the three groups. This result suggested that severe TBI did not affect the swimming speed of the mice, and motor function did not affect latency.

### Posttraumatic hypothermia increased synaptic protein expression after severe TBI

**Figure 6:**
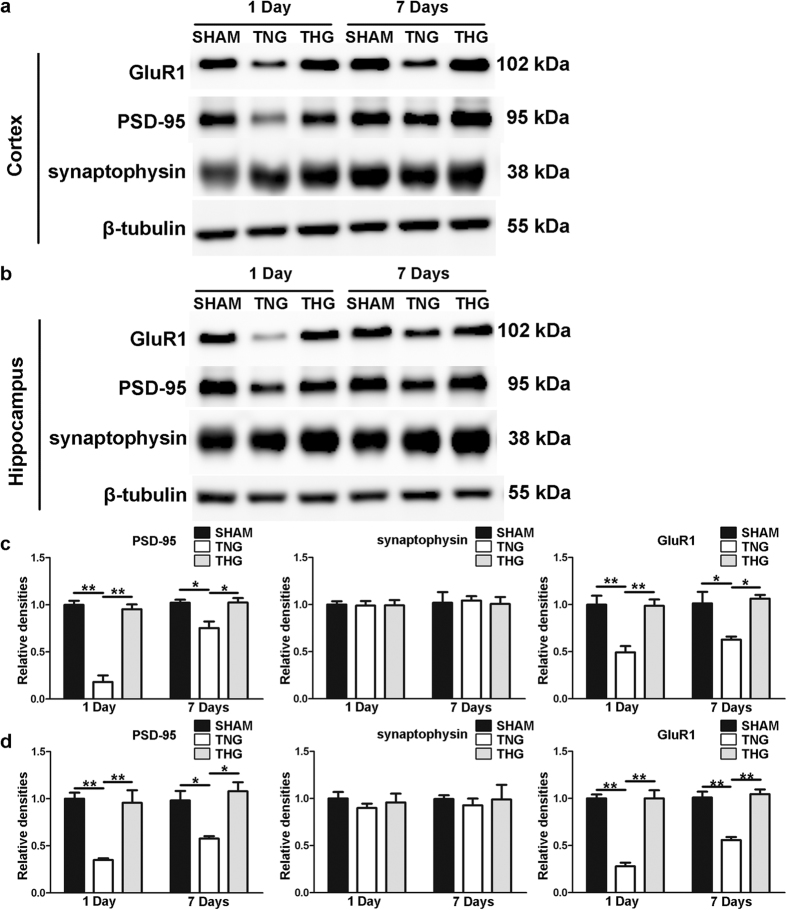
Temporal profile of synaptic proteins expression after severe TBI. (**a**) Western blot analysis was performed to evaluate the PSD-95, synaptophysin and GluR1 expression levels in the cortex 1 and 7 days after injury. (**b**) Western blot analysis was performed to evaluate the PSD-95, synaptophysin and GluR1 expression levels in hippocampus 1 and 7 days after injury. (**c,d**) Quantitative analyses of the PSD-95, synaptophysin and GluR1 expression levels in the cortex and hippocampus were performed. The results of these analyses were normalized to β-tubulin levels. The data are represented as means ± SEM and analysed using a one-way analysis of variance (ANOVA) followed by Tukey’s post hoc test, n = 10, **p < 0.01, THG or sham vs. TNG. TNG, the traumatic brain injury with normothermia group; THG, the traumatic brain injury with hypothermia group.

To further elucidate the protective effect of posttraumatic hypothermia on cortical neuron dendrites and spines after severe TBI, we assessed the changes in the expression of the synaptic proteins PSD-95, synaptophysin and GluR1 at 1 and 7 days after severe TBI in the ipsilateral cortex and hippocampus. Western blot analysis was performed to evaluate PSD-95, synaptophysin and GluR1 expression levels, and the data are shown in Fig. 6. Compared with the TNG, the THG showed significantly increased protein levels of PSD-95 and GluR1 (PSD-95 and GluR1 at 1 day in the ipsilateral cortex and hippocampus, *p* < 0.01; PSD-95 at 7 days in the ipsilateral cortex and hippocampus, *p* = 0.024 and *p* = 0.012; GluR1 at 7 days in the ipsilateral cortex and hippocampus, *p* = 0.017 and *p* < 0.01) at 1 and 7 days after severe TBI in the ipsilateral cortex and hippocampus, whereas the expression level of synaptophysin did not significantly differ among the three groups at any of the times tested. These results demonstrated that posttraumatic hypothermia increased the expression levels of PSD-95 and GluR1 in cortical and hippocampal pyramidal neurons after severe TBI, and this effect might be the result of dendrite and spine protection, leading to learning and memory function preservation.

### Posttraumatic hypothermia increased BDNF expression after severe TBI

**Figure 7:**
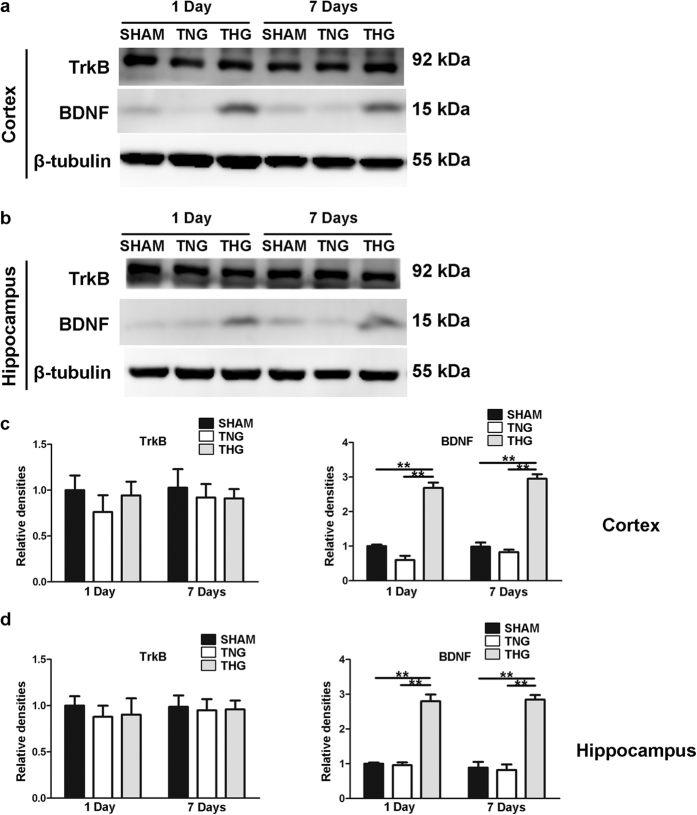
Temporal profile of BDNF and TrkB protein expression levels after severe TBI. (**a**) Western blot analysis was performed to evaluate the BDNF and TrkB expression levels in the cortex 1 and 7 days after injury. (**b**) Western blot analysis was performed to evaluate the BDNF and TrkB expression levels in the hippocampus 1 and 7 days after injury. (**c,d**) Quantitative analyses of the BDNF and TrkB expression levels in the cortex and hippocampus were performed. The results of the quantitative analyses were normalized to β-tubulin levels. The data are represented as the means ± SEM and analysed using a one-way analysis of variance (ANOVA) followed by Tukey’s post hoc test, n = 10, **p < 0.01, THG or sham vs. TNG. TNG, the traumatic brain injury with normothermia group; THG, the traumatic brain injury with hypothermia group.

To further investigate the mechanism through which induced hypothermia preserved dendrite and spine loss after severe TBI, we detected the tissue levels of BDNF and TrkB (i.e., the receptor for BDNF) at 1 and 7 days after severe TBI in the ipsilateral cortex and hippocampus. Western blot analysis showed that the protein levels of BDNF were significantly increased in the ipsilateral cortex and hippocampus of the THG at 1 and 7 days after TBI compared with that of the TNG and the sham group (*p* < 0.01); in contrast, the protein levels did not markedly differ between the TNG and the sham group (Fig. 7). In contrast, no significant changes in the TrkB protein levels were detected in the ipsilateral cortex and hippocampus of the three groups at any of the times tested. These data suggest that hypothermia-induced dendrite and spine preservation is associated with increased tissue levels of BDNF that continue for at least 7 days in the ipsilateral cortex and hippocampus after TBI.

## Discussion

Severe TBI not only causes direct brain injury but also induces secondary damage over the days immediately following. TBI causes dendrite degeneration and a significant decrease in the level of the dendrite-associated protein microtubule associated protein-2 (MAP2) at both the primary injury site and pericontused regions *in vitro*^8^,^17^. Moreover, the density of dendritic spines in the ipsilateral and contralateral cortex was dramatically reduced in mice after TBI^5^. Increasing evidence suggests that impaired dendrite and spines directly affect synaptic plasticity, which plays a critical role in neuronal function and behavioural modulation^8^,^18^. Therefore, protecting spared neural dendrites and spines after TBI might be a promising method that warrants additional research.

Mild hypothermia has been applied as an effective approach to prevent neuron death and promote cell survive after TBI^10^,^11^. Our previous research demonstrated that patients with severe TBI showed significantly improved outcomes after mild hypothermia therapy compared with a normothermia group after 1-year follow-up assessment^12^. The present study used thy1-GFP transgenic mice to explore whether hypothermia prevents dendrite and spine degeneration and improves behavioural outcomes in mice with severe TBI. Our results revealed that hypothermia markedly prevented dendrite degeneration and preserved dendrite complexity 1 and 7 days after severe TBI. The number of branches and total length of the apical dendrites in the THG were significantly increased compared with those in the TNG, and the total length of the apical dendrites did not significantly differ between the THG and the sham group 7 days after severe TBI. A Sholl analysis showed that the preserved branches of the apical dendrites were primarily concentrated 90–120 μm from the neuron soma 1 day after severe TBI as well as 90–120, 180 and 390 μm from the neuron soma 7 days after severe TBI. Posttraumatic hypothermia also protected basal dendrites from secondary injury. The total length of the basal dendrites in the THG was dramatically longer than those in the TNG 1 and 7 days after severe TBI and did not significantly differ from those in the sham group 7 days after severe TBI. However, the number of branches did not significantly differ between the THG and TNG 1 day after severe TBI, although a trend towards more branches in the THG compared with the TNG was observed. At 7 days after injury, however, the number of branches in the THG dramatically increased compared with the TNG and did not significantly differ from the sham group. The Sholl analysis showed that the preserved basal dendrite branches were concentrated primarily 30–120 μm from the neuron soma 7 days after injury. Our data showed that the effect of hypothermia on dendrite protection was persistent and continued for at least 7 days.

Dendritic spines are tiny, functional protrusions on the surface of dendrites that act as postsynaptic components to form synapses with the axonal terminal of neurons. Our study demonstrated that hypothermia affected spine protection after severe TBI. Interestingly, we found that this prevention of spine loss was regionally limited. Our spine analysis revealed that hypothermia significantly increased spine density 1–2 mm adjacent to the edge of the cavity; in contrast, no obvious difference were observed between the TNG and THG 0–1 mm from the edge of the CCI-induced cavity 1 day after severe TBI. Moreover, although the THG presented with a higher spine density than the TNG 7 days after injury, the spine density observed in the THG remained significantly lower than that of the sham group. The following reasons might account for this finding. After severe CCI, certain neurons in the nearby area suffered severe and irreversible secondary damage that hypothermia did not prevent. However, hypothermia limits the area of this irreversible damage and preserves more spines.

This study also found that posttraumatic hypothermia improves learning and memory function after severe TBI according to the MWM test. Furthermore, the sham group showed significantly better learning and memory than the THG. These data indicate that the protection of cognitive function via posttraumatic hypothermia is limited. Several modifications might be needed to optimize this treatment, such as finding the best depth and duration of hypothermia or combining this treatment with other therapies.

As a critical component of postsynaptic densities, PSD-95 functions as a N-methyl-D-aspartate (NMDA) glutamate receptor-associated protein in central synapses^19^,^20^. Several studies have reported that the NMDA glutamate receptor complex plays a central role in synaptic plasticity and memory formation^21^,^22^. Abnormal decreases in PSD-95 protein expression contribute to memory impairment *in vitro*^23^,^24^. This study found that PSD-95 expression was markedly increased in hypothermic mice after CCI compared with that measured in the TNG.

In addition to PSD-95, GluR1 is an important synaptic protein. GluR1 is a subunit of the α-amino-3-hydroxy-5-methyl-4-isoxazolepropionic acid (AMPA) glutamate receptor that mediates the most rapid excitatory synaptic transmission in the central nervous system^25^. Decreased GluR1 expression in pathological conditions is accompanied by dendritic spine loss and memory damage in rats^26^. Importantly, the present study showed that hypothermia dramatically increased PSD-95 and GluR1 protein levels after CCI, and their expression remained at normal levels. These findings might account for the effects of hypothermia regarding the promotion of learning and memory functional recovery after CCI.

Synaptophysin, one of the primary calcium-binding proteins, is distributed on the synaptic vesicle membranes. In neurons and neuroendocrine cells, synaptophysin promotes the formation of synaptic vesicles and regulates vesicular endocytosis^27^,^28^. Our results showed that the protein level of synaptophysin did not significantly differ among the three groups. This result suggests that TBI might have few effects on the formation of synaptic vesicles and biological processes that are closely related to vesicular endocytosis. Additional research is needed to investigate this possibility.

BDNF is a member of the neurotrophin family and mediates many activity-dependent processes in the mammalian brain, including axon and dendrite growth, synapse formation and plasticity, and long-term memory and learning functions^29^,^30^. The up-regulation of BDNF in the brain can stimulate synaptic protein expression and promote functional and cognitive recovery after TBI^31^,^32^. Increased levels of BDNF can also up-regulate GluR1 protein levels in 7-day *in vitro* cultured hippocampal neurons^33^ and increase PDS-95 levels in dendritic spines through dynamic microtubule invasion^34^. Moreover, the absence of BDNF in conditional BDNF-knockout mice leads to the region-specific reduction of dendritic complexity and spine density^35^. However, the direct intravenous injection of BDNF has no effect on cerebral BDNF levels because BDNF cannot be transported through the BBB *in vivo*^36^. Previous studies have shown that the induction of hypothermia during reperfusion improves neurological outcomes that might be associated with increased hippocampal levels of BDNF and TrkB (i.e., the receptor for BDNF) after asphyxia-related cardiac arrest^37^. Therefore, we hypothesized that posttraumatic hypothermia prevents dendrite degeneration and spine loss through the up-regulation of BDNF and TrkB after severe TBI. The data obtained here demonstrate that posttraumatic hypothermia can increase the cortical and hippocampal levels of BDNF at 1 and 7 days after severe TBI, whereas the expression of BDNF remained at a low level in the TNG and sham group. However, the tissue levels of TrkB did not differ among the three groups in the ipsilateral cortex and hippocampus at 1 and 7 days after injury. Increased levels of BDNF after induced hypothermia might explain the observed dendrite and spine preservation, learning and memory function promotion and increased protein levels of GluR1 and PSD-95 in the THG at 1 and 7 days after severe TBI.

In conclusion, our study demonstrated that the induction of posttraumatic hypothermia augments the tissue levels of BDNF in the ipsilateral cortex and hippocampus and dramatically prevents dendrite and spine degeneration and improves behavioural outcomes in mice after severe CCI. These findings revealed a new function of posttraumatic hypothermia in addition to reducing mortality, BBB disruption, cell death and axon injury^10^,^38^,^39^,^40^. Moreover, the mechanisms that underlie the effects of therapeutic hypothermia on dendrite and spine preservation as well as neuronal recovery promotion after TBI might be partially associated with increased levels of BDNF. However, other processes might also contribute to the beneficial effects of induced hypothermia. Additional investigations to explain these observations are warranted.

## Additional Information

**How to cite this article**: Wang, C.-f. *et al*. The Role of Posttraumatic Hypothermia in Preventing Dendrite Degeneration and Spine Loss after Severe Traumatic Brain Injury. *Sci. Rep*. **6**, 37063; doi: 10.1038/srep37063 (2016).

**Publisher’s note:** Springer Nature remains neutral with regard to jurisdictional claims in published maps and institutional affiliations.
